# A National‐Scale Assessment of Bare‐Nosed Wombat (*Vombatus ursinus*) Distribution Patterns, Using Multisource Data

**DOI:** 10.1002/ece3.73780

**Published:** 2026-05-31

**Authors:** Yuanting Jiang, Ricky‐John Spencer, Hayley J. Stannard, Julie M. Old

**Affiliations:** ^1^ School of Science, Hawkesbury Western Sydney University Penrith New South Wales Australia; ^2^ School of Agricultural, Environmental and Veterinary Sciences Charles Sturt University Wagga Wagga New South Wales Australia; ^3^ Hawkesbury Institute for the Environment Western Sydney University Penrith New South Wales Australia; ^4^ Translational Health Research Institute Western Sydney University Penrith New South Wales Australia

**Keywords:** habitat suitability, marsupial, MaxEnt, multi‐source data

## Abstract

Current understanding of bare‐nosed wombat ( *Vombatus ursinus* ) distribution has focused on specific regions, and human–wombat issues (e.g., burrowing leading to undermining fence integrity and roadkill). As their long‐term monitoring across broad spatial scales is limited by available resources, this study aimed to produce a national scale predicted habitat suitability map for the bare‐nosed wombat relying on the MaxEnt model. We analysed data from government databases the WomSAT citizen science tool, and field data from 12 sites across New South Wales. The study evaluated the major environmental characteristics (e. g. soil type and land use) that influenced the modelling and discussed occurrence in relation to these characteristics. A total of 36,210 data points reported after 2020 were used to run the model. Highly suitable habitats are mainly located in the Sydney Basin, South Eastern Corner, and South Eastern Highlands, but is scattered with limited predictions in Tasmania and the border between Victoria and South Australia. Soil and land use were identified as the key variables influencing wombat presence. Wombats are distributed in grazing land and protected areas, with a preference for Mb2, Me1 and Mw1 soil units. We encourage extending the protected area network in habitats deemed highly suitable for wombats, long‐term monitoring and implementation of effective measures to mitigate human‐wombat conflicts to support wombat conservation. When appropriate bias correction approaches are incorporated, the use of the MAXENT habitat model developed in this study could incorporate presence only, limited or uneven data sets for a range of terrestrial species to improve large‐scale distribution assessments and conservation planning.

## Graphical abstract

We used public databases and citizen science data entered into WomSAT to model and predict the distribution of bare‐nosed wombats across Australia.

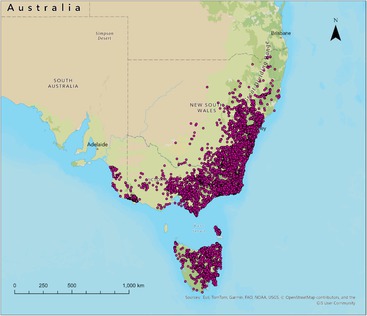

## 1 Introduction

Wombats are the largest fossorial marsupial native to Australia (Old et al. 2025). Although there were once more wombat species, there are only three remaining, the bare‐nosed wombat ( *Vombatus ursinus* ), southern hairy‐nosed wombat ( *Lasiorhinus latifrons* ), and northern hairy‐nosed wombat (*Lasiorhinus kreftii*). All species are found on mainland Australia, however two subspecies of bare‐nosed wombat (*V. u. tasmaniensis* and *V. u. ursinus*) are also found in Tasmania and the islands in Bass Strait (Thorley and Old 2020; Carver et al. 2021).

Wombats are essential to the Australian ecosystem due to their burrowing behaviour for thermoregulation and maintaining water balance (Morris et al. 2024). They are identified as ecosystem engineers because they perform an important role in maintaining surrounding ecosystem functions (Guy and Kirkpatrick 2021), including turning over soil, which enhances nitrogen cycling and water infiltration, changing soil chemistry and structure (Fleming et al. 2014). Improvements in nutrient cycling also help plant germination and growth. Moreover, their burrows provide shelter to small mammals and other animals when facing extreme threats, such as bushfire (Linley et al. 2024; Old, Hunter, and Wolfenden 2018), and the ecosystems in areas with burrows have shown a stronger resistance and recovery capacity when under threat (Linley et al. 2024).

The bare‐nosed wombat is the most widespread wombat species. Historically, they occurred across south eastern Australia, including New South Wales (NSW), the Australian Capital Territory (ACT), Victoria and Tasmania, as well as southeast Queensland, southeast South Australia, and many Bass Strait Islands (Triggs 2009; Carver et al. 2024). However, they are believed to be facing regional declines and population stress (Lunney et al. 2000). They are under threat from sarcoptic mange (Old, Sengupta, et al. 2018), which leads to skin lesions, weight loss, unusual behavioural patterns and death (Skerratt 2001; Pence and Ueckermann 2002). Roadkill and habitat loss due to human impact and climate change apply additional pressures (Nguyen et al. 2019; Matthews 2011). Subsequently, their distribution has decreased (Wallis and O'Callaghan 2018). The research on their distribution has often been limited to specific locations or threats (Murphy 2018; Mayadunnage et al. 2023; Ringwaldt et al. 2025), and a comprehensive study focusing at a national level is lacking.

To support ecological protection and conservation management, understanding the distribution of a species is essential (Mota‐Vargas and Rojas‐Soto 2012; Myers et al. 2000). By identifying biodiversity hotspots, where individuals are impacted, conservationists can systematically respond to likely challenges and in doing so, reduce conservation resources (Myers et al. 2000). However, completely understanding the distribution characteristics of a specific species is impossible because nature is dynamic and the ability of human investigation is limited (Hortal et al. 2015). Hence, the data used by scientists are incomplete and possibly lack representativeness, which can lead to misidentifying species status and predicted changes, hence leading to inefficient and ineffective conservation outcomes. The development of biodiversity informatics (the application of computer technology to support collection, management, analysis and visualisation of biodiversity data) provides a shared, sustainable platform that combines the whole research community and citizen science (Hardisty et al. 2013), offering the possibility of reducing these risks and integrating more complete data, by enabling the integration of multiple data sources.

In Australia there are many biodiversity data platforms including extensive data on bare‐nosed wombat occurrence. The databases hosted by governments, such as NSW BioNet and the Tasmanian Natural Values Atlas, collect and store fieldwork and monitoring records uploaded by scientists (Environment and Heritage 2025; Department of Natural Resources and Environment 2025). These data generally have higher accuracy but are limited in collecting data regarding home range and time observed. In contrast, citizen science tools rely on contributions made by citizens, which can effectively cover wider geographic areas and support updates at any time (Miller‐Rushing et al. 2012; Dickinson et al. 2010). To monitor wombats, WomSAT was developed based on the NSW Department of Primary Industry's FeralScan website in 2015 (Skelton et al. 2019). It can be downloaded and used on smartphones or directly accessed via the website (Skelton et al. 2019). Users can upload wombat, burrow, or treatment information, including photos, specific GPS coordinates, time, sex and health status (whether alive or dead and sarcoptic mange score). In addition, the Atlas of Living Australia, as a national biodiversity infrastructure, sources biodiversity data from multiple resources, including scientists and citizen science tools, and operates based on the Darwin Core standards (Atlas of Living Australia 2025), which provides a stable, standardised framework of terms and definitions for biological diversity data (Darwin Core Maintenance Group 2026).

Using multi‐source data brings both opportunities and risks. Increasing the amount of available data and their spatial coverage promotes more systematic research and large‐scale distribution prediction (Miller‐Rushing et al. 2012; Dickinson et al. 2010). However, their consistency and reliability are questioned when conducting a biodiversity investigation, for example, as records are commonly collected around roads, towns, or other easily accessible areas; spatial biases are unavoidable (Kadmon et al. 2004; Amano et al. 2016). When studies focus on temporal patterns, using opportunity data can introduce temporal biases due to inconsistent work effort (Isaac et al. 2014; Amano et al. 2016). More importantly, citizen science data are more likely to have quality issues than scientific data (Hecht and Spicer Rice 2015), which can lead to misidentification and misdirected effort. Therefore, scientists should consciously identify biases and plan targeted strategies when incorporating data into models and ground‐truth the results through further focused surveys (Bonney et al. 2009).

Although a large amount of bare‐nosed wombat data is stored in multiple online open‐access databases, there is still a lack of systematic assessment of these sources to determine the distribution of bare‐nosed wombats at a national level. This study collected comprehensive bare‐nosed wombat occurrence data from multiple open‐access datasets, such as the Atlas of Living Australia and NSW BioNet, to build a species distribution model using MaxEnt. We aimed to predict distribution and hotspots at the national level, as well as the spatial pattern of occurrence driven by key environmental variables, which provide a stronger foundation for future monitoring and management of bare‐nosed wombats nationally.

## 2 Method

### 2.1 Data Collection and Cleaning

Online occurrence reports were collected from two open‐access citizen science databases and six Australian state environment department databases (Table 1) between the 22nd and 27th March 2024 using the search term ‘ *Vombatus ursinus*’. An extra dataset included treatment records spanning May 2021 to May 2025 provided by the Wombat Protection Society of Australia Incorporated (WPSA). All datasets were downloaded and entered into Microsoft Excel using the key fields of ID, date, latitude and longitude.

**TABLE 1:** Open‐access databases used for collecting wombat occurrence data.

Database	Total number of records downloaded	Date downloaded	Link
Atlas of Living Australia	104,855	22nd March 2024	https://ala.org.au/
WomSAT	22,976	22nd March 2024	https://www.womsat.org.au/womsat/
NSW BioNet	83,746	25th March 2024	https://atlas.bionet.nsw.gov.au/
Victorian Biodiversity Atlas	17,930	26th March 2024	https://vba.biodiversity.vic.gov.au/vba/#/
Natural Values Atlas (TAS)	16,720	25th March 2024	https://www.naturalvaluesatlas.tas.gov.au/
NatureMaps (SA)	218	25th March 2024	http://spatialwebapps.environment.sa.gov.au/naturemaps/?locale=en‐us&viewer=naturemaps
ACT Wildlife Atlas	145	25th March 2024	https://www.gbif.org/dataset/8810b12b‐7a24‐403e‐8b44‐bf3848badb35
WildNet (QLD)	139	27th March 2024	https://apps.des.qld.gov.au/species‐search/

Data were initially filtered in Microsoft Excel by removing records having no report date or GPS coordinates. Duplicate data, defined as having the same latitude, longitude, and report date, were removed using the ‘Remove Duplicate’ tool. As different GPS devices and environments may affect GPS coordinate accuracy (Tomaštík Jr. et al. 2016), we further excluded records when both their latitude and longitude difference was < 0.0001 with the same date.

The cleaned data was uploaded into Google Earth Pro for spatial validation. We removed the data if they occurred far away from the potential habitat range, such as in the ocean and outside Australia. Since WomSAT data included both bare‐nosed wombat and hairy‐nosed wombat information (https://www.womsat.org.au/womsat/), photographs accompanying WomSAT records were cross‐checked and records of species that were not bare‐nosed wombats excluded.

### 2.2 Field Data Collection

**FIGURE 1:**
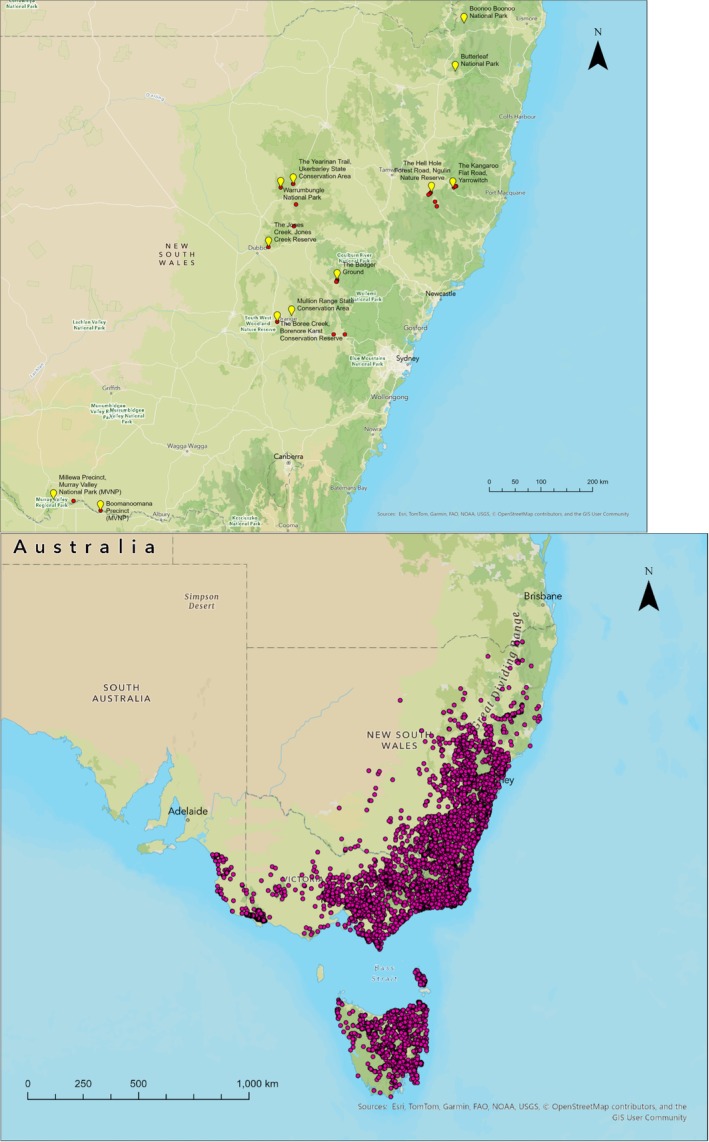
(a) Map indicating field site locations (yellow) and positive wombat sightings, burrows and signs (red) obtained in this study. (b) Raw occurrence records of bare‐nosed wombat used for modelling.

Field surveys were conducted across 12 sites in New South Wales between November 2024 and May 2025 (Figure 1a; Table S1). The sites were chosen based on the perceived limits of the BNW distribution obtained through the online databases. Transect surveys were undertaken by driving a 4WD vehicle at a speed less than 10 km per hour (Stannard et al. 2021). A minimum of two observers was included. The 100‐W halogen spotlights (Powa‐Beam: PL‐145, 12 V) were used to scan both sides of the road when driving at night. Short‐range walks were also used when driving was not feasible due to reduced visibility and accessibility issues, and any wombat scats and burrows observed were recorded. Opportunistic observations of roadkill whilst travelling between survey sites were also recorded.

Recorded information included the GPS coordinates of where the wombat was located and the time. When sightings occurred from within the vehicle, the GPS coordinates of the vehicle, the distance to the individual, and the compass bearing to the individual were recorded. The geographic coordinates of wombats were subsequently calculated in Google Earth Pro (Stannard et al. 2021).

### 2.3 Environmental Data Collection and Pre‐Processing

Environmental variables were chosen based on those used in previous studies investigating wombats (Swinbourne et al. 2021; Mayadunnage et al. 2023; Fryett et al. 2025). Collected environmental variables (Table 2) included bioclimatic variables (Fick and Hijmans 2017), elevation data (Fick and Hijmans 2017), Euclidean distance to water, soil type (National Resource Information Centre 1991), catchment scale land use data (ABARES 2024) and vegetation type (Department of Climate Change‚ Energy‚ the Environment and Water 2025b). The standard bioclimatic (bio 1–19) and elevation layers were downloaded from WorldClim version 2 at a resolution of 30 s (Fick and Hijmans 2017). Soil type layer included the information on both the soils' landscapes and component soils (National Resource Information Centre 1991).

**TABLE 2:** Environmental variables used for MaxEnt modelling.

Environmental variable	References
Bio2—Mean Diurnal Range (Mean of monthly (max temp—min temp))	Fick and Hijmans (2017)
Bio4—Temperature Seasonality (standard deviation ×100)	Fick and Hijmans (2017)
Bio8—Mean Temperature of Wettest Quarter	Fick and Hijmans (2017)
Bio9—Mean Temperature of Driest Quarter	Fick and Hijmans (2017)
Bio15—Precipitation Seasonality (Coefficient of Variation)	Fick and Hijmans (2017)
Bio17—Precipitation of Driest Quarter	Fick and Hijmans (2017)
Bio18—Precipitation of Warmest Quarter	Fick and Hijmans (2017)
Euclidean distance to water—hydrology lines	Crossman and Li (2015a)
Euclidean distance to water—hydrology polygons	Crossman and Li (2015b)
Elevation	Fick and Hijmans (2017)
Land use	ABARES (2024)
Vegetation subgroups	Department of Climate Change‚ Energy‚ the Environment and Water (2025a, 2025b)
Soil type	National Resource Information Centre (1991)

Euclidean distance to water was calculated in ArcGIS Pro using the ‘Euclidean Distance’ tool. Input hydrological surface rasters were downloaded from Geoscience Australia (Crossman and Li 2015a, 2015b) involving surface hydrology lines (watercourses, canals, pipelines, etc.) and surface hydrology polygons (swamps, reservoirs, etc.), which forms the entire flow path network of Australia.

Pearson correlation coefficients were calculated between continuous variables to remove highly correlated variables (Merow et al. 2013). Continuous variables included bioclimatic variables, elevation and Euclidean distance to water. One of two variables were kept if a pair of variables showed a correlation coefficient > 0.7. Criteria for inclusion were based on the ecological relevance and Mayadunnage et al. (2023) and Fryett et al. (2025). Final variables retained for modelling are listed in Table 2, and layers were prepared as ASCII files for MaxEnt analyses.

All environmental layers were converted to raster and aligned at the same snap raster, coordinate system (World Geodetic System 1984, used in GPS—EPSG: 4326), cell size (X/Y = 0.008333 degree) and extent in ArcGIS Pro. Extent was defined by the spatial coverage of the soil type layer (National Resource Information Centre 1991), which covers all terrestrial landmasses of Australia, including the mainland, Tasmania and offshore islands.

### 2.4 Bias File Preparation and the Definition of the Accessible Area

Although our data was collected from multiple databases with most uploaded from scientist and scientific projects, some citizen science records from WomSAT and the Atlas of Living Australia were included. This data may be affected by human population density, where high search effort can easily lead to sampling bias (Phillips et al. 2009). To reduce the influence of bias in the model, a buffered minimum convex polygon (MCP) and a Kernel density layer were added (Santori et al. 2018; Mayadunnage et al. 2023), which can apply lower weighting to regions with lower presences when selecting background points (Xu et al. 2024). Bias files were generated using the ‘Sample by Buffered MCP’ tool and the ‘Gaussian Kernel Density of Sampling Localities’ tool by the SDMtoolbox Pro v0.9.1 (Brown et al. 2017). All bias layers were aligned to the same extent and projection to environmental variable layers and were exported as ASCII grids for running model.

The accessible area is the area the species can realistically occur based on dispersal, ecological and historical constraints. In this study, accessible area was used for post hoc visualisation and interpretation. Due to the study objective of predicting species distribution at a national scale, it was not used in modelling and background sampling. The accessible area was mapped in ArcGIS Pro according to the Interim Biogeographic Regionalisation for Australia (IBRA) (Department of Climate Change‚ Energy‚ the Environment and Water 2025a). IBRA regions were selected using both historical and recent occurrence records. However, records located in the boundary between two IBRA regions were excluded from IBRA selection when they resulted in the inclusion of a single isolated IBRA region without additional records. A 50 km buffer was applied to account for dispersal uncertainty. The accessible area for mainland Australia and Tasmania with offshore islands was mapped separately to reflect the geographic isolation among subspecies of bare‐nosed wombat.

### 2.5 Modelling Setting and Evaluation

We developed three models using MaxEnt 3.4.4, including the original model, the background model with a buffered MCP bias file, and the density model with a Kernel density bias file, based on previous studies and guidelines (Brown et al. 2017, Boria et al. 2014, Young et al. 2011, Santori et al. 2018, Mayadunnage et al. 2023). We randomly set 25% data as a test file to evaluate the performance of each model (Young et al. 2011). The setting for background points remained at a default of 10,000. Fifteen subsample replicates with random seeds were run to ensure the model's stability (Young et al. 2011). To ensure the model reached convergence, we set maximum iterations to 5000. The convergence threshold remained as the default setting with 0.00001. Models were run using default feature classes which allow automatic selection of linear, quadratic, hinge, product and threshold features based on sample size. Soil type, land use and vegetation type layers were defined as categorical variables in MaxEnt. The logistic output was interpreted as the relative habitat suitability for bare‐nosed wombat and exported as ASCII grids.

To find a more parsimonious model and reduce the risk of model overfitting, models were run with three regularisation multipliers (1, 1.5 and 2) (Phillips 2010), therefore, we obtained nine outputs (Table S2). Model performance was evaluated using the average area under the curve (AUC) and the average omission and predicted area. If the mean omission rate and predicted omission rate are close, the model has good performance (Young et al. 2011; Elith et al. 2011); and an AUC value close to 1 usually indicates a reliable model.

Moreover, we assessed the relative importance of environmental variables based on the estimates of relative contributions and the Jackknife test of variable importance (Mayadunnage et al. 2023). The estimates of relative contribution consisted of the percent contribution which indicates the relative contribution of variables to the gain increase and permutation importance which shows the uniqueness of each variable (Elith et al. 2011).

In this paper, we only present the predicted map and Jackknife test output of the best performing model. The final map was generated by ArcGIS Pro where relative suitable values were classified as ‘not suitable’, ‘moderately suitable’, ‘suitable’ and ‘highly suitable’. The minimum raster cell (threshold) was determined using the 10th percentile training presence logistic threshold with the average of 0.2305 across 15 replicates. The 10th percentile training presence threshold was adopted as it balances omission error with realistic expected prevalence in disturbed species ranges (Liu et al. 2016). To define other classes, the values over 0.2305 were partitioned into three equal intervals, which were 0.2305–0.487 (moderately suitable), 0.487–0.7435 (suitable) and 0.7435–1 (highly suitable).

To avoid overestimates of model performance due to structured data and to assess model transferability, we applied a non‐random cross‐validation for evaluating models (Wenger and Olden 2012). Since the spatial extent of the study area was substantially larger than the sampled regions, an environmental cross‐validation was run using the ENMeval 2.0.5.2 package in RStudio 4.5.2 (Muscarella et al. 2014). Occurrence data were partitioned into four folds based on multivariate environmental similarity. All parameter settings were kept consistent with the original MaxEnt configuration.

Model performance on field data was evaluated using a threshold‐based binomial test by comparing the proportion of field data located on regions where MaxEnt predicted suitability above threshold against background point expectations. A total of 1000 background points were randomly selected in ArcGIS Pro within the extent consistent with the MaxEnt setting of the final model. The binomial test was performed in RStudio 4.5.2.

### 2.6 Habitat Preference Analysis

Additional descriptive analyses were undertaken to evaluate the habitat preference of bare‐nosed wombat across the important environmental variables identified by the Jackknife test. The value of the environmental layer was extracted to each occurrence point using the ‘Extract Multi Values to Point’ tool in ArcGIS Pro. A CSV table was generated using the ‘Summary Statistics’ tool, containing a category column and frequency within each category, and the statistic type was set to ‘Count’. To visualise the preference of occurrence, bar charts were generated and the y‐axis was explained as the distribution for each classification (%).

## 3 Results

A total of 246,729 reports were downloaded, covering the period from January 1760 to March 2024. After cleaning duplicates and errors, 142,731 reports remained, including ALA (*n* = 57,729), WomSAT (*n* = 15,998), WildNet (*n* = 137), Victorian Biodiversity Atlas (*n* = 16,425), Natural Values Atlas (*n* = 11,535), BioNet (*n* = 40,569), ACT Wildlife Atlas (*n* = 142) and NatureMaps (*n* = 196). However, we only used online occurrence data that was reported after 2020 (*n* = 33,891) during model runs to ensure that the final result reflected the current distribution of bare‐nosed wombats (Department of Climate Change‚ Energy‚ the Environment and Water 2023). Moreover, a further 3128 records provided by WPSA was included.

Thirty‐nine field records of bare‐nosed wombat were included which were obtained during our field surveys. All live individuals were recorded at the Badger Ground (*n* = 6). Nine roadkill records, ten scat and fourteen burrows were also included. One burrow located at the Kangaroo Flat was filled with water.

When running the models, 848 records were removed because environmental data were not available at those geographic locations. Therefore, a total of 36,210 data were included in the final model runs (Figure 1b).

### 3.1 Modelling of Bare‐Nosed Wombat Distribution

We compared three types of models under different regularisation multipliers (1, 1.5 and 2). The original model and the density model with a multiplier of 1 presented severe overfitting. The predicted output only relied on one single environmental variable. Therefore, these outputs were excluded from the final model selection. Both the kernel density model and the original model ran with higher regularisation multipliers (1.5 and 2) and gained high AUC (more than 0.96) and low standard deviation (< 0.0015); however, the outputs showed a clear mismatch between the mean omission rate on test data and the predicted omission rate. The mean omission was significantly higher than that predicted at lower cumulative thresholds and lower at higher thresholds. In addition, none of the original models and the density models converged before reaching maximum iteration (5000).

In contrast, the background models presented more stable and better calibrated performance. The mean omission rate was close to the predicted omission rate in all background model outputs. At the regularisation multiplier of 1, the average training AUC across 15 replicates was 0.8904 and the average test AUC was 0.8882 (SD = 0.0032, 95% CI: 0.8866–0.8898, *n* = 15). The model achieved an average AUC of 0.7668 (SD = 0.0729) and average Continuous Boyce Index (CBI) of 0.771 (SD = 0.2236) under environmental cross‐validation. For the 1.5 regularisation multiplier model, the average training AUC was 0.8934 and the average test AUC was 0.8898 (SD = 0.0032, 95% CI: 0.8882–0.8914, *n* = 15). Environmental cross‐validation resulted in an average AUC of 0.7622, which was lower than that of the 1 regularisation multiplier model, but variability across folds was reduced, with a standard deviation of 0.0664. The average CBI was 0.7685 (SD = 0.2090). When the regularisation multiplier was 2, MaxEnt model performance slightly declined (training AUC = 0.8815, test AUC = 0.8796, SD = 0.0033, 95% CI: 0.8779–0.8813, *n* = 15), with lower performance under environmental cross‐validation (AUC = 0.7600, SD = 0.0637; CBI = 0.7553, SD = 0.1926). A summary of the key outputs for the nine models is available in Table S2.

**FIGURE 2:**
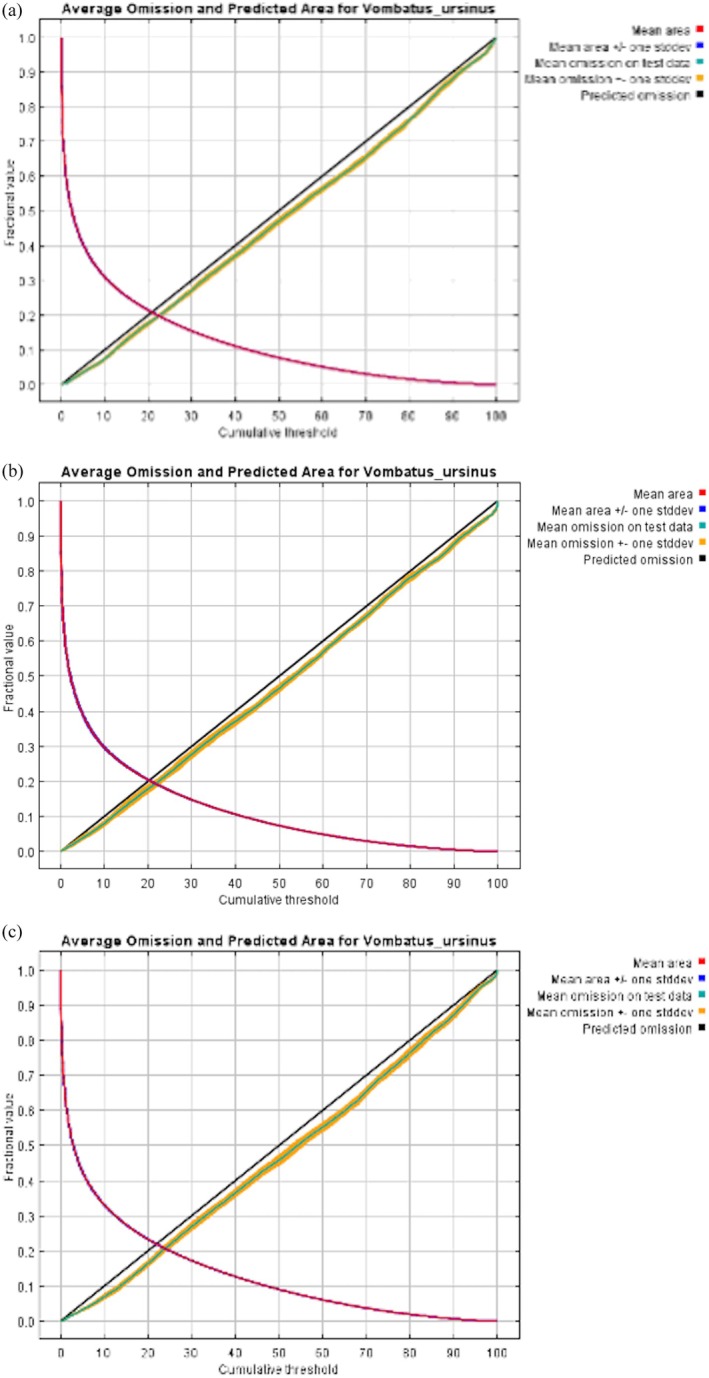
Average omission and Predicted Area curves for three regularisation multipliers (background model); (a) rm. = 1; (b) rm. = 1.5; (c) rm. = 2. The mean omission ± one standard deviation (yellow lines) for rm. = 1 and rm. = 1.5 more closely match the mean omission on test data (green line) compared to rm. = 2. Of all figures, the mean omission curve for rm. = 1.5 model showed the closest alignment with the predicted omission curve (black line).

**FIGURE 3:**
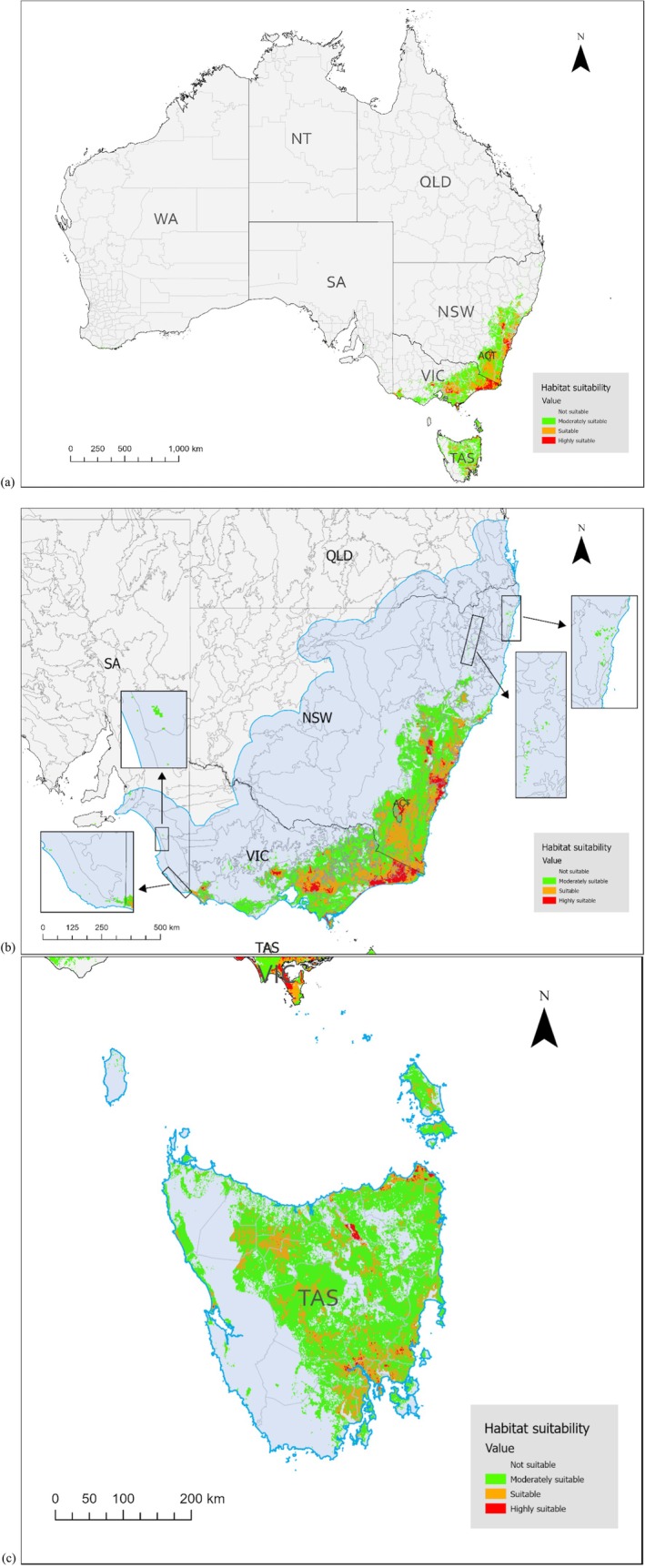
(a) National scale habitat suitability map for the bare‐nosed wombat (logistic output—background model with 1.5 regularisation multipliers); (b) Suitability map masked post hoc by the accessible area for mainland individuals (*V. u. hirsutus*); (c) Suitability map masked post hoc by the accessible area for the Tasmanian subspecies (*V. u. tasmaniensis* and *V. u. ursinus*). The accessible area is coloured light blue with a blue outline. Highly suitable habitats (> 0.7435) are coloured with red; suitable habitats (0.487–0.7435) were coloured with orange; and moderately suitable habitats (0.2305–0.487) were coloured with green.

Based on MaxEnt performance, environmental cross‐validation output, and the closely matched mean omission curve (Figure 2), the logistic output of the background model (1.5 regularisation multiplier) was explained as the relative habitat suitability and regions with higher probability values were identified as predicted hotspots of bare‐nosed wombat distribution (Figure 3a). The map was masked post hoc using accessible area and was shown separately for mainland Australia (Figure 3b) and Tasmania (Figure 3c). The Tasmanian prediction was visualised independently to reflect suitability patterns associated with *V. u. tasmaniensis* and *V. u. ursinus*.

Highly suitable habitats (value > 0.7435) only appeared in NSW, ACT, Victoria and Tasmania, and occurred as spatially scattered patches (Figure 3a). Suitable habitat (value > 0.487) extended into the southeastern corner of South Australia, and the northeastern corner of NSW. A few small moderately suitable patches (probability between 0.2305–0.487) were predicted on North Stradbroke Island in Queensland, the central coastal region of South Australia, as well as the southwestern coast of Western Australia; however, these regions lack verified records of bare‐nosed wombats.

Under the 10th percentile training presence threshold (0.2305), 19 out of 39 field records (*p̂* = 0.4872) were successfully predicted by MaxEnt and 284 of 1000 background points were predicted (*p̂* = 0.284). The proportion of field records was significantly higher than expected under random background points (*p* = 0.0057; 95% CI: 0.3472–1.0000).

### 3.2 Spatial Pattern

**FIGURE 4:**
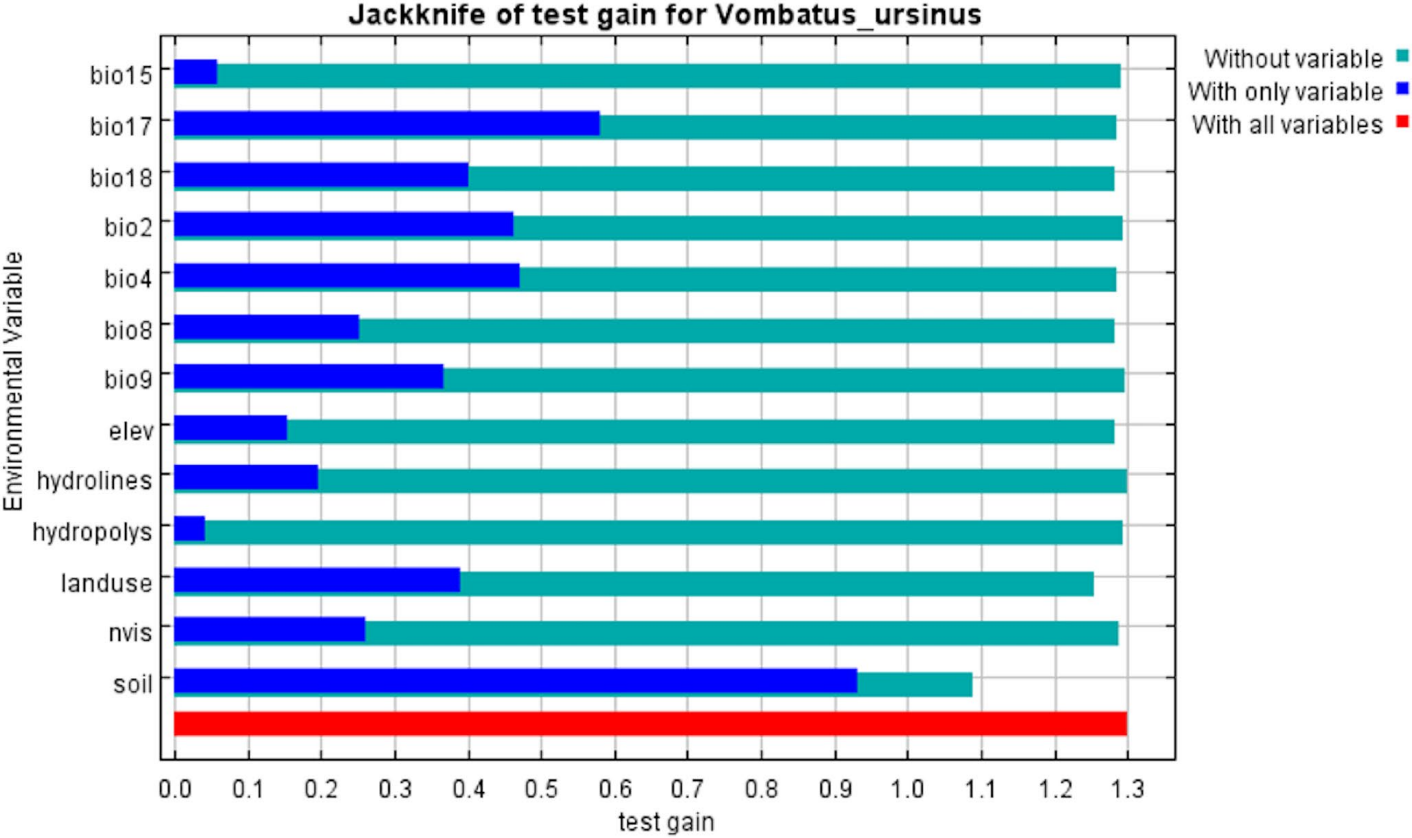
Results of the jackknife test of variable importance using test gain. Refer to Table 2 for the description of the environmental variables.

Percent contribution (Table 3) confirmed that soil type was the most influential predictor with the contribution of 48.9% when predicting the occurrence of bare‐nosed wombat, followed by land use (16.8%) and temperature seasonality (bio4; 11.6%). The highest permutation importance variable (Table 3) was Bio18 (precipitation of warmest quarter), accounting for 41.2%, but it showed relatively low contribution (5.1%). The Jackknife test confirmed that omitting soil type and land use led to significant reduction in test gain (Figure 4), proving their importance as an explanatory value. Therefore, due to the importance of these two categories, we calculated the percentage of occurrence for each soil type and land use.

**TABLE 3:** Estimates of relative contributions of the environmental variables to the Maxent model.

Variable	Unit	Value	Percent contribution	Permutation importance
Soil type	Categories	3046	48.9	19.7
Land use	Categories	188	16.8	3.8
Bio4	%	74.9–684.3	11.6	8.1
Bio17	mm	0–898	5.9	4.9
Bio18	mm	10–2595	5.1	41.2
Bio2	°C	1.60–17.5	4.1	1.1
Vegetation subgroups	Categories	84	3.2	0.4
Bio15	mm	5.9–145.9	2.1	1.3
Bio9	°C	2.4–29.2	1	3.7
Elevation	mm	−43–2144	0.4	3.9
Bio8	°C	−0.6–32.8	0.4	10.8
Euclidean distance to water—hydrology polygons	mm	0–1135504.25	0.4	0.9
Euclidean distance to water—hydrology lines	mm	0–1159892.125	0	0

*Note:* Refer to Table 2 for the description of the environmental variables.

**FIGURE 5:**
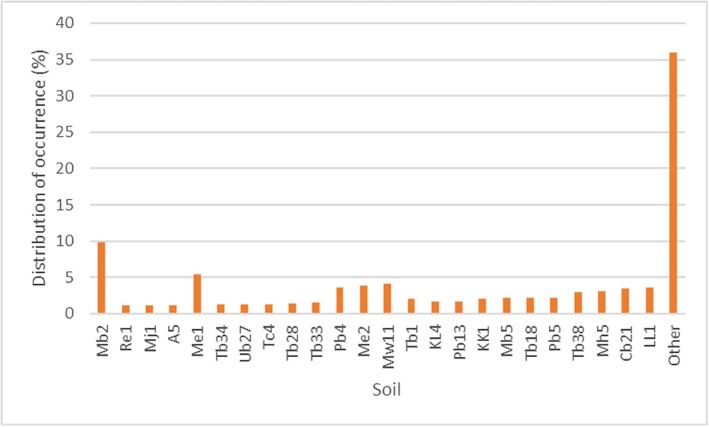
Bare‐nosed wombat distribution within each soil unit. Refer to Table S3 for the description of the soil units. Soil unit layers were downloaded from the National Resource Information Centre (1991).

Bare‐nosed wombats can be found in a wide range of soil classifications (*n* = 381). Records of bare‐nosed wombats were most frequent in soil classification units Mb2, Me1 and Mw1 (Figure 5, Table S3).

**FIGURE 6:**
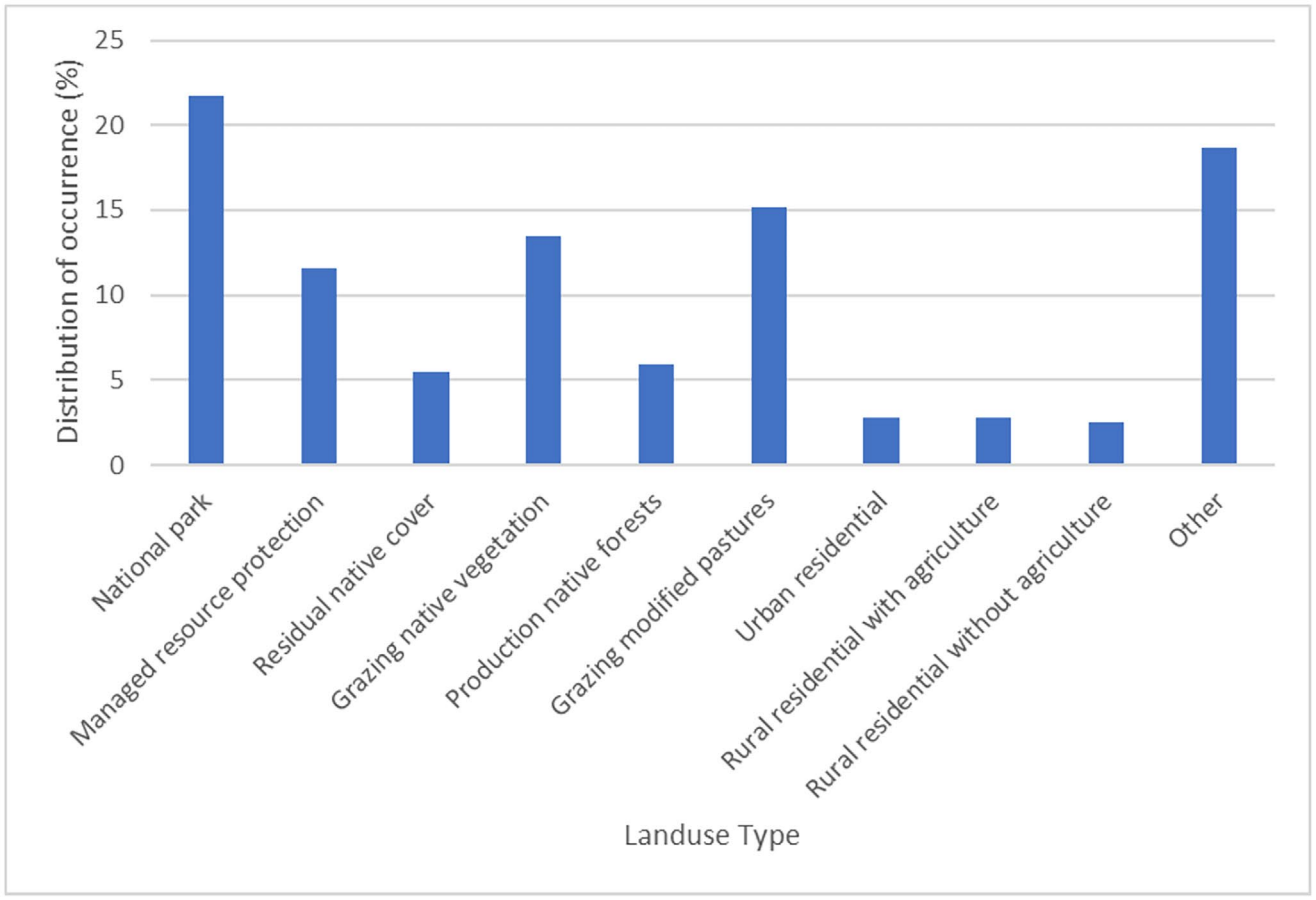
Occurrence reported within each land use. Land use type layers were downloaded from the ABARES (2024).

Bare‐nosed wombats were reported across 93 different land use classifications with the highest occurrence in national parks (21.69%; Figure 6).

## 4 Discussion

### 4.1 Bare‐Nosed Wombat Distribution

The logistic output of MaxEnt is the relative habitat suitability rather than the true probability (Elith et al. 2011), meaning the result does not represent the absolute probability of occurrence. The model predicted that highly suitable habitat occurred in southeastern Australia, including NSW, the ACT, Victoria and Tasmania. Some habitats with lower suitability extended further south to southeastern South Australia, and north to the North Coast of NSW and the border with Queensland. Generally, our predicted distribution range aligns with previous studies on bare‐nosed wombat distribution (Thorley and Old 2020; Wallis and O'Callaghan 2018; Evans 2008; Knoblauch et al. 2023), as well as studies limited to states, New South Wales (Lunney et al. 2000; Matthews et al. 2011), Victoria (Coulson and Cripps 2026) and Tasmania (Driessen et al. 2022) and specific issues such as roadkill (Mayadunnage et al. 2023) and sarcoptic mange (Mayadunnage et al. 2024; Fryett et al. 2025). On the basis of the Interim Biogeographic Regionalisation for Australia (Department of Climate Change‚ Energy‚ the Environment and Water 2025a), we identified that hotspots occur in the Sydney Basin, South Eastern Highlands, South East Corner, South East Coastal Plain and Victoria Midlands (Figure S1). However, some small highly suitable habitats were also located in Tasmania, the NSW North Coast and the Naracoorte Coastal Plain within Victoria, which indicated a more fragmented trend. Land use changes, diseases, roadkill, illegal shooting and climate change are all possible driving factors resulting in the fragmentation and contraction (Roger et al. 2007; Old, Sengupta, et al. 2018; Environment and Heritage 2024a; Thorley and Old 2020). Some records may also be the result of human‐assisted movement as suggested by Williams and Menkhorst (1995) and Coulson and Cripps (2026). Also, given that the occurrence data used for model development were restricted to records after 2020, the predicted fragmentation patterns may partially reflect the effects of the 2019–2020 megafires (Ward et al. 2020). Although many bare‐nosed wombats are thought to survive fire events by sheltering in burrows, bushfires can still lead to indirect effects, such as changes in vegetation structure and reduced resource availability (Miritis et al. 2024). However, as bushfires represent dynamic disturbances, their effects are not fully captured by the static environmental variables used in this study. Investigating historical records may also indicate trends and distribution over time and identify key threatened processes such as fire.

The model also predicted some scattered patches as moderately suitable habitats on the southwestern coast of Western Australia, the central coastal region of South Australia and North Stradbroke Island in Queensland, but no occurrences have been reported in these areas. The reason for this is that the extent of environmental layers we used covered all of Australia, and MaxEnt predicted suitable habitats based on the correlations across the entire country (Phillips et al. 2006). The current understanding of bare‐nosed wombat distribution has confirmed that they did not extend to Western Australia and the central coastal region of South Australia near Adelaide (Triggs 2009). Moreover, because we included the whole of Australia in the model, performance declined after environmental cross‐validation, which suggests that the model has limited transferability and that predictions outside the accessible area should be considered cautiously. Predicted areas beyond the accessible area could reflect climatic analogues rather than biologically accessible habitats. It is not evidence of real range extension but potential false positives arising from environmental similarities. Therefore, despite the environmental characteristics in these areas being consistent with habitats suitable for the bare‐nosed wombat, they should be excluded.

Environmental cross‐validation was conducted to support environmental transferability of the model. However, spatial block cross‐validation cannot be applied because it assumed that both the environmental layer and presence data layer have the same extent (Valavi et al. 2018). Since the environmental layer covered a much broader extent than the presence data layer in our study, using spatial cross‐validation likely produced many empty folds, further affecting the accuracy of evaluation. We recommend that future analyses clip the extent to the potential maximum range identified from this study with a buffer area and further apply spatial cross‐validation to assess spatial transferability of the model.

### 4.2 Environmental Variables

The Jackknife test output indicated that soil type (Table S3) was the most important environmental variable explaining wombat distribution, which is associated with the wombat's burrowing behaviour. Burrows are essential for wombats and assist with regulating body temperature and water balance, as well as providing shelter from predators and extreme weather events (Morris et al. 2024). Therefore, suitable soil for burrow construction is crucial. Our descriptive analysis of wombat occurrence data confirmed that the landscape where wombats and burrows are most abundant is slopes, hills and undulating ridges. A key driving factor leading to this landscape preference is flooding, as burrows on the flat areas were easily subject to flooding (Triggs 2009). Field surveys confirmed that most of the major burrows were dug on slopes which would minimise the risk of flooding (Triggs 2009).

Borchard et al. (2008) argued that wombats prioritise the difficulty of burrowing over reduced flooding risk, as more wombat burrows were discovered along higher‐order stream banks. The bare‐nosed wombat was commonly distributed in soil units of Mb2, Me1, Mw1, which primarily contain acid leached yellow or red earths, brown or red friable earths and uniform loamy soils, but was rarely distributed in the units containing large amounts of ironstone gravels and boulders (Table S3). After analysing the population on the Blowering Reservoir foreshores within Kosciuszko National Park, Roger et al. (2007) also believed that the bare‐nosed wombat can adapt to various soil types, but it usually avoided burrowing in barren or rocky areas.

The soil layer was downloaded from the Digital Atlas of Australia Soils (National Resource Information Centre 1991), which is a nationally consistent dataset using uniform soil unit nomenclature. However, this product conflates both soil type and landscape information, which may lead to ambiguity in ecological interpretation. In addition, although we did not analyse soil bulk density, it is also an important factor that influences wombat occurrence in summer in the subalpine zone of the Snowy Mountains (Matthews et al. 2010). Bare‐nosed wombats typically avoided areas where soil bulk density was low because it represents very wet sites, which emphasised the importance of soil drainage to wombats. Therefore, we suggest that future studies incorporate more detailed and separated soil and landscape variables; factors such as slope, soil texture, drainage capacity, soil depth and bulk density may provide more ecologically meaningful predictors than generalised soil classifications alone.

In this study the distribution of bare‐nosed wombats was also influenced by land use. Occurrence records included in the study were generally concentrated in grazing land, but more than half of those records were in grazing modified pastures instead of grazing land with native vegetation. Agricultural landscapes provide more stable and abundant food resources so wombat populations in pastures have higher feeding efficiency than those living in forests (McIlroy 1973; Taylor 1993; Buchan and Goldney 1998), but the distribution of burrows is limited by the remaining vegetation areas (Skerratt et al. 2004). Additionally, the increased wombat activity in grazing land has significantly raised the risk of human‐wildlife conflicts (McIlroy 1973; O'Brien 2019; Swinbourne et al. 2017). Although the NSW *Biodiversity Conservation Act 2016* provides legal protection to wombats, some landowners still hold negative attitudes towards them. Wombats damage farm fences and other infrastructure; sometimes their burrows also pose risks of tripping and falling to livestock (Environment and Heritage 2024a, 2024b).

Bare‐nosed wombats were also commonly reported in national parks and managed resource protected areas, which reflects the importance of establishing protected areas for maintaining wombat populations. However, a report on Maria Island National Park confirmed that wombats were still under pressure from resource competition with other species, such as the Forester kangaroo ( *Macropus giganteus tasmaniensis* ) and the continuous threat from sarcoptic mange (Ingram 2015). The report also mentioned that wombats have been detected in the scat of the Tasmanian devil ( *Sarcophilus harrisii* ), although their population on Maria Island has not been affected. Moreover, as protected areas are not isolated regions from human activity, such areas cannot protect wombats from roadkill (Roger et al. 2012). Therefore, the survival status of wombat populations within the protected areas needs to be continuously assessed. Records in urban residential areas and rural residential areas showed that they have relative adaptability to fragmented and complex habitats. However, this adaptability is accompanied by high risks of roadkill and human impact, as evidenced in previous studies (Mayadunnage et al. 2023).

Vegetation subgroup and distance to water made little contribution to the modelling in our study. This difference is interesting, as multiple studies have described the preference of distribution within vegetation types (Guy and Kirkpatrick 2021; Knoblauch et al. 2023; Roger et al. 2007; Borchard and Eldridge 2011) and Roger et al. (2007) also emphasised the contribution of proximity to watercourses. The reason for this difference unclear, but a reasonable assumption is that vegetation and distance to water variables were collinear with other categorical variables. Since Pearson correlation coefficients were only used to exclude highly correlated continuous variables from modelling (Merow et al. 2013), potential redundancy among categorical variables was not assessed. A study conducted in Kangaroo Valley, NSW confirmed that the abundance of bare‐nosed wombat burrows was positively correlated with shrub cover, but this association was reversed in native shrub, with the increased cover the abundance declined (Borchard et al. 2008). The abundance was also positively associated with stream order and vegetation width (Borchard et al. 2008). We recommend future studies discuss redundancy and reevaluate the contribution of vegetation and distance to water and include shrub cover, stream order and vegetation width as additional variables.

### 4.3 Modelling and Limitations

In our study, the original model produced the highest training AUC values and test AUC values, but the mean omission rates substantially deviated from the predicted omission rates, and the models were trained to rely on a single variable. Such patterns demonstrated the limitation of presence‐only data. Due to the lack of absence data, MaxEnt randomly selected background points, as pseudo‐absence, from the full layer extent (Phillips et al. 2006). Choosing background points across a wide area increased commission error, as background points were more likely to overlap with suitable but unoccupied or biogeographically isolated habitats, which can lead to overfitting (Brown et al. 2017). Therefore, controlling the background points using bias files is essential when modelling with presence‐only data.

Although bias correction strategies can improve model performance to some extent, presence‐only models remain inherently limited compared with models incorporating true absence data, which have previously been shown to generate substantially different spatial prediction patterns (Brizuela‐Torres et al. 2024). WomSAT has begun collecting absence records, providing opportunities for future studies to incorporate these data into generalised linear models and other presence‐absence models. Future studies should include targeted surveys in remote areas and consider incorporating additional correction methods related to accessibility, such as variables of distance to roads and distance to settlements, to better account for roadside and human activity bias. These approaches may help distinguish true habitat suitability from patterns driven by uneven observer accessibility, particularly in low‐accessibility regions where increasing survey effort is logistically difficult.

The performance of the kernel density model was also poor, showing overfitting. We suggest the main reason for this overfitting was due to the inclusion of citizen science data. Reports from citizen scientists were generally distributed along roads or in areas with higher human activity, resulting in a different sampling intensity compared to the low accessibility areas (Amano et al. 2016; Skelton et al. 2019). As the kernel density layer reduces the impact of sampling bias on model estimation by weighting high‐density areas (Xu et al. 2024), the model was likely affected by the high density of citizen science data and was trained heavily dependent on environmental characteristics of high sampling effort areas, including land use types. In addition, when some regions with low sampling occurrence represent real low density or sparsely occupied habitats, there is a risk of introducing bias into the model.

The buffered MCP layer was considered the most suitable bias file in our study, as the background model showed a more robust performance, which was consistent with the finding from a study on roadkill prediction using WomSAT data (Mayadunnage et al. 2023). Since the buffered MCP bias file approximates the accessible area rather than sampled space, it does not weight regions based on sampling effort. Of the three models with different regularisation multipliers, the model with 1.5 multiplier showed the best omission curves and calibration, which aligns with the published MaxEnt guidelines (Radosavljevic and Anderson 2014; Phillips 2010; Young et al. 2011). They proposed that higher regularisation is conducive to reducing the risks of overfitting and producing a more regular model. However, the consistency of the omission curve declined after the multiplier increased to 2, whilst the predicted distribution map showed a larger colouring range with blurred habitat boundaries. As in previous studies, excessive regularisation multipliers produced an overly smoothed model, which reduced the quality and overall discernibility (Radosavljevic and Anderson 2014). Therefore, attempting multiple regularisation multipliers to select an appropriate value was highly encouraged (Elith et al. 2011).

In this study, we did not clip the modelling extent according to a defined accessible area. Although such an approach led to over prediction in some regions, such as Western Australia, these areas can be reasonably excluded based on post hoc accessible area and knowledge of current wombat distribution. Considering the limited understanding of the accurate maximum dispersal boundaries (Triggs 2009; Thorley and Old 2020), our approach ensured inclusion of all potentially suitable edge habitats.

Although a threshold‐based binomial test was conducted to support the model performance on predicting field observations, only 39 data was too small relative to the online data. Conducing independent modelling likely introduces higher uncertainty. We also recommend that future analyses include additional targeted surveys in predicted highly suitable habitats lacking records and along the range edges for prospective validation.

## 5 Conclusion

This study is the first comprehensive national scale investigation of the recent distribution pattern of the bare‐nosed wombat using multi‐source data (from citizen science tools, government databases and fieldwork), providing a scientific basis for wombat conservation. The study indicated that the buffered minimum convex polygon bias file offered the most stable prediction, avoiding overfitting observed in the original models and the kernel models. By analysing 36,210 occurrence records in MaxEnt, soil type was identified as the most influential factor, followed by land use. Our study confirmed that the highly suitable habitats for bare‐nosed wombats are scattered in south eastern Australia, particularly within the Sydney Basin, South Eastern Corner and South Eastern Highlands. Due to the preference of habitat selection on grazing land and protected areas, formulating appropriate strategies to reduce human pressure and establishing targeted protected areas where wombats can be continuously monitored are recommended.

Next steps should include the use of the maximum range and increased field work on the range edge for further validation. Additional separate soil layers such as bulk density, depth, and texture could also be incorporated.

The MaxEnt habitat model developed in this study, with appropriate bias correction approaches, could be applicable for other terrestrial species, particularly species for which only presence data or limited and uneven coverage exist, including citizen science data sets, such as those used in this study. Integrating traditional and non‐traditional data could improve environmental modelling for both threatened and non‐threatened species as well as pest species. It could also be used to identify high conservation areas for multiple species through the identification of overlapping areas in modelled habitat. Hence, the inclusion of multi‐source datasets may provide a practical strategy for improving large‐scale distribution assessments and supporting conservation planning efforts for species where traditional survey data remains limited.

## Author Contributions

**Yuanting Jiang:** conceptualization (equal), writing – original draft (equal). **Ricky‐John Spencer:** conceptualization (equal). **Hayley J. Stannard:** conceptualization (equal), writing – original draft (equal). **Julie M. Old:** conceptualization (equal), writing – original draft (equal).

## Funding

Funding was provided through donations to WomSAT and RTF from Western Sydney University.

## Ethics Statement

Field surveys were approved by the Charles Sturt University Animal Ethics Committee (AEC) with approval number A24409, with reciprocal approval from the Western Sydney University AEC (A16599).

## Conflicts of Interest

The authors declare no conflicts of interest.

## Supporting information


**Figure S1:** (a) IBRA bioregions used for geographic orientation in identifying hotspots; (b) IBRA map version 7.1 from the Department of Climate Change‚ Energy‚ the Environment and Water (2025a).
**Table S1:** Study sites where fieldwork was conducted.
**Table S2:** Key output from the nine models.
**Table S3:** Official descriptions for the main soil type contributing to the modelling (National Resource Information Centre 1991).

## Data Availability

All data is publicly available and/or provided in the Supporting Information.

## References

[ece373780-bib-0001] ABARES . 2024. Catchment Scale Land Use of Australia – Update December 2023 Version 2. Australian Bureau of Agricultural and Resource Economics and Sciences.

[ece373780-bib-0002] Amano, T. , J. D. Lamming , and W. J. Sutherland . 2016. “Spatial Gaps in Global Biodiversity Information and the Role of Citizen Science.” Bioscience 66, no. 5: 393–400.

[ece373780-bib-0003] Atlas of Living Australia . 2025. https://www.ala.org.au/about‐ala/.

[ece373780-bib-0004] Bonney, R. , C. B. Cooper , J. Dickinson , et al. 2009. “Citizen Science: A Developing Tool for Expanding Science Knowledge and Scientific Literacy.” Bioscience 59, no. 11: 977–984.

[ece373780-bib-0005] Borchard, P. , and D. J. Eldridge . 2011. “The Geomorphic Signature of Bare‐Nosed Wombats ( *Vombatus ursinus* ) and Cattle ( *Bos taurus* ) in an Agricultural Riparian Ecosystem.” Geomorphology 130, no. 3–4: 365–373.

[ece373780-bib-0006] Borchard, P. , J. McIlroy , and C. McArthur . 2008. “Links Between Riparian Characteristics and the Abundance of Common Wombat ( *Vombatus ursinus* ) Burrows in an Agricultural Landscape.” Wildlife Research 35, no. 8: 760–767.

[ece373780-bib-0201] Boria, R. A. , L. E. Olson , S. M. Goodman , and R. P. Anderson . 2014. “Spatial Filtering to Reduce Sampling Bias Can Improve the Performance of Ecological Niche Models.” Ecological modelling 275: 73–77.

[ece373780-bib-0007] Brizuela‐Torres, D. , J. Elith , G. Guillera‐Arroita , and N. J. Briscoe . 2024. “Dealing With Sampling Bias and Inferring Absence Data to Improve Distribution Models of a Widely Distributed Vulnerable Marsupial.” Austral Ecology 49, no. 1: e13474.

[ece373780-bib-0008] Brown, J. L. , J. R. Bennett , and C. M. French . 2017. “SDMtoolbox 2.0: The Next Generation Python‐Based GIS Toolkit for Landscape Genetic, Biogeographic and Species Distribution Model Analyses.” PeerJ 5: e4095. 29230356 10.7717/peerj.4095 PMC5721907

[ece373780-bib-0009] Buchan, A. , and D. C. Goldney . 1998. “The Common Wombat *Vombatus ursinus* in a Fragmented Landscape.” In Wombats, edited by R. T. Wells and P. A. Pridmore . Surrey Beatty and Sons in association with the Royal Zoological Society of South Australia.

[ece373780-bib-0010] Carver, S. , M. Charleston , G. Hocking , R. Gales , and M. M. Driessen . 2021. “Long‐Term Spatiotemporal Dynamics and Factors Associated With Trends in Bare‐Nosed Wombats.” Journal of Wildlife Management 85, no. 3: 449–461.

[ece373780-bib-0011] Carver, S. , G. L. Stannard , and A. M. Martin . 2024. “The Distinctive Biology and Characteristics of the Bare‐Nosed Wombat ( *Vombatus ursinus* ).” Annual Review of Animal Biosciences 12: 135–160. 37738454 10.1146/annurev-animal-021022-042133

[ece373780-bib-0012] Coulson, G. , and J. K. Cripps . 2026. “The Bare‐Nosed Wombat: A Review of Biology and Management in Victoria.” Proceedings of the Royal Society of Victoria 138: RS25013.

[ece373780-bib-0013] Crossman, S. , and O. Li . 2015a. Surface Hydrology Lines (National). Geoscience Australia.

[ece373780-bib-0014] Crossman, S. , and O. Li . 2015b. Surface Hydrology Polygons (National). Geoscience Australia.

[ece373780-bib-0015] Darwin Core Maintenance Group . 2026. “Darwin Core [Online].” https://dwc.tdwg.org.

[ece373780-bib-0016] Department of Climate Change‚ Energy‚ the Environment and Water . 2023. “On‐Ground Surveys and Data for Referred Actions Under the EPBC Act.” https://www.dcceew.gov.au/environment/epbc/advice/surveys‐and‐data. Accessed 21 September 2025.

[ece373780-bib-0017] Department of Climate Change‚ Energy‚ the Environment and Water . 2025a. Australia's Bioregions (IBRA). Australian Government.

[ece373780-bib-0018] Department of Climate Change‚ Energy‚ the Environment and Water . 2025b. National Vegetation Information System Data Products. Australian Government.

[ece373780-bib-0019] Department of Natural Resources and Environment . 2025. Natural Values Atlas. Tasmanian Government.

[ece373780-bib-0020] Dickinson, J. L. , B. Zuckerberg , and D. N. Bonter . 2010. “Citizen Science as an Ecological Research Tool: Challenges and Benefits.” Annual Review of Ecology, Evolution, and Systematics 41: 149–172.

[ece373780-bib-0021] Driessen, M. M. , E. Dewar , S. Carver , C. Lawrence , and R. Gales . 2022. “Conservation Status of Common Wombats in Tasmania II: Population Distribution and Trends, and the Incidence and Significance of Roadkill.” Pacific Conservation Biology 28, no. 2: 115–123.

[ece373780-bib-0022] Elith, J. , S. J. Phillips , T. Hastie , M. Dudík , Y. E. Chee , and C. J. Yates . 2011. “A Statistical Explanation of MaxEnt for Ecologists.” Diversity and Distributions 17, no. 1: 43–57.

[ece373780-bib-0023] Environment and Heritage . 2024a. Living With Wombats. NSW Government.

[ece373780-bib-0024] Environment and Heritage . 2024b. Wombats. NSW Government.

[ece373780-bib-0025] Environment and Heritage . 2025. About BioNet Atlas. NSW Government.

[ece373780-bib-0026] Evans, M. C. 2008. “Home Range, Burrow‐Use and Activity Patterns in Common Wombats ( *Vombatus ursinus* ).” Wildlife Research 35, no. 5: 455–462.

[ece373780-bib-0027] Fick, S. E. , and R. J. Hijmans . 2017. “ worldclim 2: New 1‐Km Spatial Resolution Climate Surfaces for Global Land Areas.” International Journal of Climatology 37, no. 12: 4302–4315.

[ece373780-bib-0028] Fleming, P. A. , H. Anderson , A. S. Prendergast , M. R. Bretz , L. E. Valentine , and G. E. S. Hardy . 2014. “Is the Loss of Australian Digging Mammals Contributing to a Deterioration in Ecosystem Function?” Mammal Review 44, no. 2: 94–108.

[ece373780-bib-0029] Fryett, E. R. , C. N. Subasinghe , J. M. Old , and H. J. Stannard . 2025. “Determining Environmental Factors That Influence the Occurrence of Sarcoptic Mange in Bare‐Nosed Wombats ( *Vombatus ursinus* ) Using Citizen Science Data.” Transboundary and Emerging Diseases 2025, no. 1: 6264097. 41383392 10.1155/tbed/6264097 PMC12695404

[ece373780-bib-0030] Guy, T. R. , and J. B. Kirkpatrick . 2021. “Environmental Associations and Effects of Disturbances by Common Wombats in Alpine Tasmania.” Austral Ecology 46, no. 8: 1392–1403.

[ece373780-bib-0031] Hardisty, A. , D. Roberts , and The Biodiversity Informatics Community . 2013. “A Decadal View of Biodiversity Informatics: Challenges and Priorities.” BMC Ecology 13, no. 1: 16. 23587026 10.1186/1472-6785-13-16 PMC3843378

[ece373780-bib-0032] Hecht, J. , and E. Spicer Rice . 2015. “Citizen Science: A New Direction in Canine Behavior Research.” Behavioural Processes 110: 125–132. 25444773 10.1016/j.beproc.2014.10.014

[ece373780-bib-0033] Hortal, J. , F. de Bello , J. A. F. Diniz‐Filho , T. M. Lewinsohn , J. M. Lobo , and R. J. Ladle . 2015. “Seven Shortfalls That Beset Large‐Scale Knowledge of Biodiversity.” Annual Review of Ecology, Evolution, and Systematics 46: 523–549.

[ece373780-bib-0034] Ingram, J. 2015. “The Current Status of Wombat Populations on Maria Island National Park.”

[ece373780-bib-0035] Isaac, N. J. , A. J. van Strien , T. A. August , M. P. de Zeeuw , and D. B. Roy . 2014. “Statistics for Citizen Science: Extracting Signals of Change From Noisy Ecological Data.” Methods in Ecology and Evolution 5, no. 10: 1052–1060.

[ece373780-bib-0036] Kadmon, R. , O. Farber , and A. Danin . 2004. “Effect of Roadside Bias on the Accuracy of Predictive Maps Produced by Bioclimatic Models.” Ecological Applications 14, no. 2: 401–413.

[ece373780-bib-0037] Knoblauch, W. , S. Carver , M. M. Driessen , R. Gales , and S. A. Richards . 2023. “Abundance and Population Growth Estimates for Bare‐Nosed Wombats.” Ecology and Evolution 13, no. 9: e10465. 37674647 10.1002/ece3.10465 PMC10477484

[ece373780-bib-0038] Linley, G. D. , W. L. Geary , C. J. Jolly , et al. 2024. “Wombat Burrows Are Hotspots for Small Vertebrates in a Landscape Subject to Gigafire.” Journal of Mammalogy 105, no. 4: 752–764. 39081267 10.1093/jmammal/gyae034 PMC11285166

[ece373780-bib-0039] Liu, C. , G. Newell , and M. White . 2016. “On the Selection of Thresholds for Predicting Species Occurrence With Presence‐Only Data.” Ecology and Evolution 6, no. 1: 337–348. 26811797 10.1002/ece3.1878 PMC4716501

[ece373780-bib-0040] Lunney, D. , A. Curtin , D. Ayers , et al. 2000. The Threatened and Non‐Threatened Vertebrate Fauna of New South Wales: An Ecological Interpretation. NSW National Parks and Wildlife Service, Environmental and Heritage Monograph.

[ece373780-bib-0041] Matthews, A. 2011. “Climate Change Influences on the Distribution and Resource Use of Common Wombats *Vombatus ursinus* in the Snowy Mountains Australia.”

[ece373780-bib-0042] Matthews, A. , D. Lunney , M. Crowther , J. V. Bryant , and A. Shannoon . 2011. “The Distribution and Community Perceptions of the Common Wombat in New South Wales, National Wombat Conference, Albury.”

[ece373780-bib-0043] Matthews, A. , P. G. Spooner , D. Lunney , K. Green , and N. I. Klomp . 2010. “The Influences of Snow Cover, Vegetation and Topography on the Upper Range Limit of Common Wombats *Vombatus ursinus* in the Subalpine Zone, Australia.” Diversity and Distributions 16, no. 2: 277–287.

[ece373780-bib-0044] Mayadunnage, S. , H. J. Stannard , P. West , and J. M. Old . 2024. “Spatial and Temporal Patterns of Sarcoptic Mange in Wombats Using the Citizen Science Tool, WomSAT.” Integrative Zoology 19, no. 3: 387–399. 37865949 10.1111/1749-4877.12776

[ece373780-bib-0045] Mayadunnage, S. , H. J. Stannard , P. West , J. M. Old , and R. Goldingay . 2023. “Identification of Roadkill Hotspots and the Factors Affecting Wombat Vehicle Collisions Using the Citizen Science Tool, WomSAT.” Australian Mammalogy 45, no. 1: 53–61.

[ece373780-bib-0046] McIlroy, J. C. 1973. Aspects of the Ecology of the Common Wombat, *Vombatus ursinus* (Shaw, 1800). Australian National University (Australia).

[ece373780-bib-0047] Merow, C. , M. J. Smith , and J. A. Silander Jr. 2013. “A Practical Guide to MaxEnt for Modeling Species' Distributions: What It Does, and Why Inputs and Settings Matter.” Ecography 36, no. 10: 1058–1069.

[ece373780-bib-0048] Miller‐Rushing, A. , R. Primack , and R. Bonney . 2012. “The History of Public Participation in Ecological Research.” Frontiers in Ecology and the Environment 10, no. 6: 285–290.

[ece373780-bib-0049] Miritis, V. , C. R. Dickman , D. G. Nimmo , and T. S. Doherty . 2024. “After the ‘Black Summer’ Fires: Faunal Responses to Megafire Depend on Fire Severity, Proportional Area Burnt and Vegetation Type.” Journal of Applied Ecology 61: 63–75.

[ece373780-bib-0050] Morris, S. D. , C. N. Johnson , B. W. Brook , and M. R. Kearney . 2024. “Seasonal and Depth‐Dependent Thermoregulatory Benefits of Burrows for Wombats – The Largest Burrowing Marsupials.” Journal of Thermal Biology 125: 103961. 39405735 10.1016/j.jtherbio.2024.103961

[ece373780-bib-0051] Mota‐Vargas, C. , and O. R. Rojas‐Soto . 2012. “The Importance of Defining the Geographic Distribution of Species for Conservation: The Case of the Bearded Wood‐Partridge.” Journal for Nature Conservation 20, no. 1: 10–17.

[ece373780-bib-0052] Murphy, M. J. 2018. “Records of the Common Wombat *Vombatus ursinus* (Shaw, 1800) in the Pilliga Forest, Northern Inland New South Wales.” Victorian Naturalist 135, no. 3: 64–71.

[ece373780-bib-0053] Muscarella, R. , P. J. Galante , M. Soley‐Guardia , et al. 2014. “ENMeval: An R Package for Conducting Spatially Independent Evaluations and Estimating Optimal Model Complexity for Maxent Ecological Niche Models.” Methods in Ecology and Evolution 5, no. 11: 1198–1205.

[ece373780-bib-0054] Myers, N. , R. A. Mittermeier , C. G. Mittermeier , G. A. B. da Fonseca , and J. Kent . 2000. “Biodiversity Hotspots for Conservation Priorities.” Nature 403, no. 6772: 853–858. 10706275 10.1038/35002501

[ece373780-bib-0055] National Resource Information Centre . 1991. Digital Atlas of Australian Soils. National Resource Information Centre.

[ece373780-bib-0056] Nguyen, H. K. , M. W. Fielding , J. C. Buettel , and B. W. Brook . 2019. “Habitat Suitability, Live Abundance and Their Link to Road Mortality of Tasmanian Wildlife.” Wildlife Research 46, no. 3: 236–246.

[ece373780-bib-0057] O'Brien, C. 2019. Understanding the Causes of Human‐Wombat Conflict and Exploring Non‐Lethal Damage Mitigation Strategies for the Southern Hairy‐Nosed Wombat ( *Lasiorhinus latifrons* ). School of Biological Sciences, University of Adelaide.

[ece373780-bib-0058] Old, J. , H. Stannard , J. C. Z. Woinarski , and A. A. Burbidge . 2025. “ *Vombatus ursinus* .” The IUCN Red List of Threatened Species. 10.2305/IUCN.UK.2025-1.RLTS.T40556A258654868.en.

[ece373780-bib-0059] Old, J. M. , N. E. Hunter , and J. Wolfenden . 2018. “Who Utilises Bare‐Nosed Wombat Burrows?” Australian Zoologist 39, no. 3: 409–413.

[ece373780-bib-0060] Old, J. M. , C. Sengupta , E. Narayan , and J. Wolfenden . 2018. “Sarcoptic Mange in Wombats‐A Review and Future Research Directions.” Transboundary and Emerging Diseases 65, no. 2: 399–407. 29150905 10.1111/tbed.12770

[ece373780-bib-0061] Pence, D. , and E. Ueckermann . 2002. “Sarcoptic Mange in Wildlife.” Revue Scientifique et Technique‐Office International Des Epizooties 21, no. 1: 385–398. 11974622

[ece373780-bib-0062] Phillips, S. 2010. “A Brief Tutorial on Maxent.”

[ece373780-bib-0063] Phillips, S. J. , R. P. Anderson , and R. E. Schapire . 2006. “Maximum Entropy Modeling of Species Geographic Distributions.” Ecological Modelling 190, no. 3: 231–259.

[ece373780-bib-0064] Phillips, S. J. , M. Dudík , J. Elith , et al. 2009. “Sample Selection Bias and Presence‐Only Distribution Models: Implications for Background and Pseudo‐Absence Data.” Ecological Applications 19, no. 1: 181–197. 19323182 10.1890/07-2153.1

[ece373780-bib-0065] Radosavljevic, A. , and R. P. Anderson . 2014. “Making Better Maxent Models of Species Distributions: Complexity, Overfitting and Evaluation.” Journal of Biogeography 41, no. 4: 629–643.

[ece373780-bib-0066] Ringwaldt, E. , S. Richards , and S. Carver . 2025. “A Landscape View of Sarcoptic Mange in Bare‐Nosed Wombats Across New South Wales.”

[ece373780-bib-0067] Roger, E. , G. Bino , and D. Ramp . 2012. “Linking Habitat Suitability and Road Mortalities Across Geographic Ranges.” Landscape Ecology 27, no. 8: 1167–1181.

[ece373780-bib-0068] Roger, E. , S. W. Laffan , and D. Ramp . 2007. “Habitat Selection by the Common Wombat ( *Vombatus ursinus* ) in Disturbed Environments: Implications for the Conservation of a 'common' Species.” Biological Conservation 137, no. 3: 437–449.

[ece373780-bib-0069] Santori, C. , R.‐J. Spencer , J. U. Van Dyke , and M. B. Thompson . 2018. “Road Mortality of the Eastern Long‐Necked Turtle ( *Chelodina longicollis* ) Along the Murray River, Australia: An Assessment Using Citizen Science.” Australian Journal of Zoology 66, no. 1: 41–49.

[ece373780-bib-0070] Skelton, C. J. , A. S. Cook , P. West , R. J. Spencer , and J. M. Old . 2019. “Building an Army of Wombat Warriors: Developing and Sustaining a Citizen Science Project.” Australian Mammalogy 41, no. 2: 186–195.

[ece373780-bib-0071] Skerratt, L. 2001. “Management of Sarcoptic Mange in Wombat Populations.” Proceedings of Veterinary Conservation Biology: Wildlife Health and Management in Australasia 45: 271–275.

[ece373780-bib-0072] Skerratt, L. F. , J. H. L. Skerratt , S. Banks , R. Martin , and K. Handasyde . 2004. “Aspects of the Ecology of Common Wombats ( *Vombatus ursinus* ) at High Density on Pastoral Land in Victoria.” Australian Journal of Zoology 52, no. 3: 303–330.

[ece373780-bib-0073] Stannard, H. J. , J. Wolfenden , E. M. Hermsen , B. T. Vallin , N. E. Hunter , and J. M. Old . 2021. “Incidence of Sarcoptic Mange in Bare‐Nosed Wombats ( *Vombatus ursinus* ).” Australian Mammalogy 43, no. 1: 85–95.

[ece373780-bib-0074] Swinbourne, M. , D. Taggart , and B. Ostendorf . 2021. “The Population Status of Southern Hairy‐Nosed Wombats ( *Lasiorhinus latifrons* ). II. Landscape Factors Affecting Distribution and Abundance.” Australian Mammalogy 43: 54–65.

[ece373780-bib-0075] Swinbourne, M. , D. Taggart , D. Peacock , and B. Ostendorf . 2017. “Historical Changes in the Distribution of Hairy‐Nosed Wombats (*Lasiorhinus* spp.): A Review.” Australian Mammalogy 39, no. 1: 1–16.

[ece373780-bib-0076] Taylor, R. J. 1993. “Observations on the Behaviour and Ecology of the Common Wombat *Vombatus ursinus* in Northeast Tasmania.” Australian Mammalogy 16, no. 1: 1–7.

[ece373780-bib-0077] Thorley, R. K. , and J. M. Old . 2020. “Distribution, Abundance and Threats to Bare‐Nosed Wombats ( *Vombatus ursinus* ).” Australian Mammalogy 42, no. 3: 249–256.

[ece373780-bib-0078] Tomaštík, J., Jr. , J. Tomaštík Sr. , Š. Saloň , and R. Piroh . 2016. “Horizontal Accuracy and Applicability of Smartphone GNSS Positioning in Forests.” Forestry: An International Journal of Forest Research 90, no. 2: 187–198.

[ece373780-bib-0079] Triggs, B. 2009. Wombats. CSIRO Publishing.

[ece373780-bib-0080] Valavi, R. , J. Elith , J. J. Lahoz‐Monfort , and G. Guillera‐Arroita . 2018. “blockCV: An R Package for Generating Spatially or Environmentally Separated Folds for k‐Fold Cross‐Validation of Species Distribution Models.” bioRxiv. 10.1101/357798.

[ece373780-bib-0081] Wallis, R. , and E. O'Callaghan . 2018. “Historical Reports of Common (Bare‐Nosed) Wombats *Vombatus ursinus* in the Warrnambool Area, Victoria.” Victorian Naturalist 135, no. 6: 178–180.

[ece373780-bib-0082] Ward, M. , A. I. T. Tulloch , J. Q. Radford , et al. 2020. “Impact of 2019–2020 Mega‐Fires on Australian Fauna Habitat.” Nature Ecology & Evolution 4: 1321–1326. 32690905 10.1038/s41559-020-1251-1

[ece373780-bib-0083] Wenger, S. J. , and J. D. Olden . 2012. “Assessing Transferability of Ecological Models: An Underappreciated Aspect of Statistical Validation.” Methods in Ecology and Evolution 3, no. 2: 260–267.

[ece373780-bib-0084] Williams, L. L. , and P. W. Menkhorst . 1995. “Common Wombat.” Mammals of Victoria. Distribution, Ecology and Conservation 45: 82–83.

[ece373780-bib-0085] Xu, Q. , X. Wang , J. Yi , and Y. Wang . 2024. “Bias Correction in Species Distribution Models Based on Geographic and Environmental Characteristics.” Ecological Informatics 81: 102604.

[ece373780-bib-0086] Young, N. , L. Carter , and P. Evangelista . 2011. A MaxEnt Model v3. 3.3 e Tutorial (ArcGIS v10). Natural Resource Ecology Laboratory, Colorado State University and the National Institute of Invasive Species Science.

