# Supplementary material for: A National‐Scale Assessment of Bare‐Nosed Wombat (Vombatus ursinus) Distribution Patterns, Using Multisource Data
Source: Ecol Evol. 2026 May 31;16(6):e73780. doi: 10.1002/ece3.73780 (PMC13240152; doi:10.1002/ece3.73780)
Supplement: Supplementary file 1 — Figure S1: (a) IBRA bioregions used for geographic orientation in identifying hotspots; (b) IBRA map version 7.1 from the Department of Climate Change‚ Energy‚ the Environment and Water (2025a). Table S1: Study sites where fieldwork was conducted. Table S2: Key output from the nine models. Table S3: Official descriptions for the main soil type contributing to the modelling (National Resource Information Centre 1991). [file ECE3-16-e73780-s001.docx]

Supplementary Materials

1.
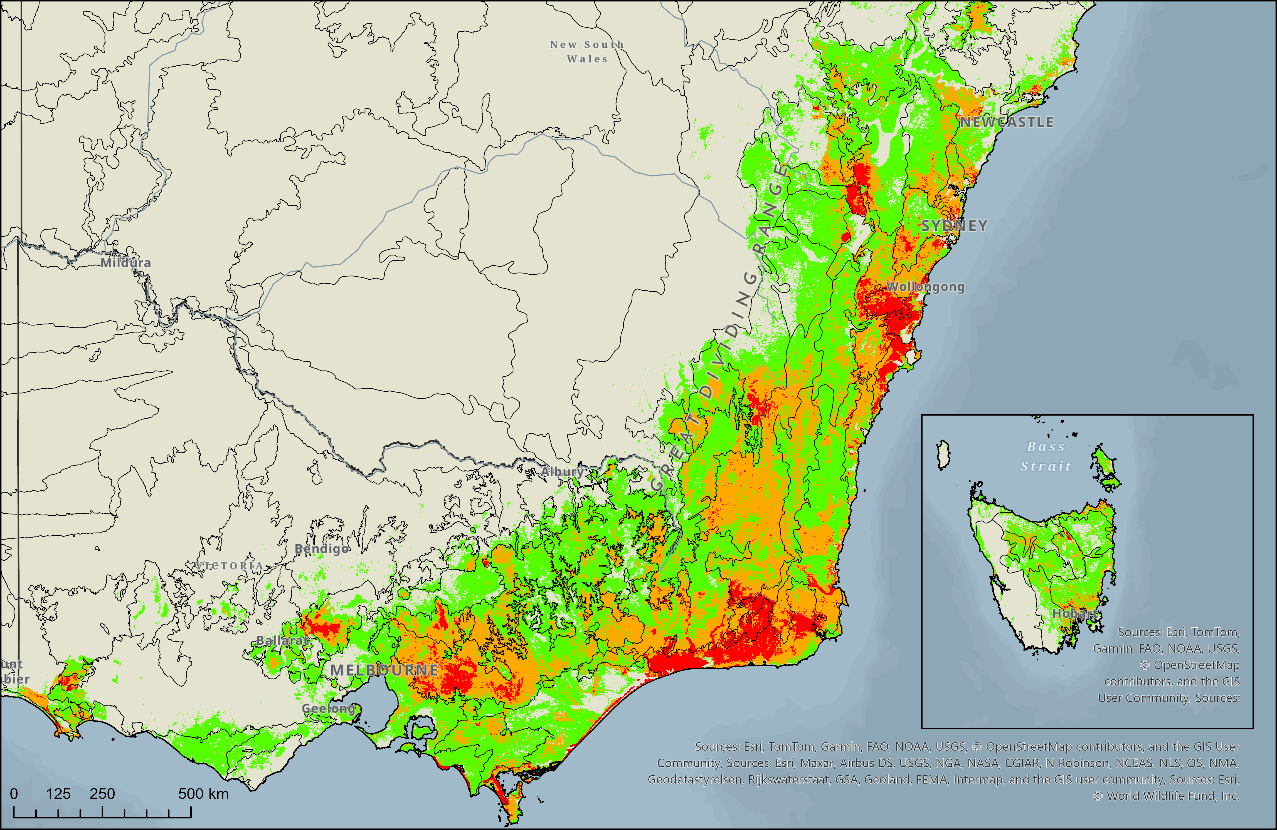

2.
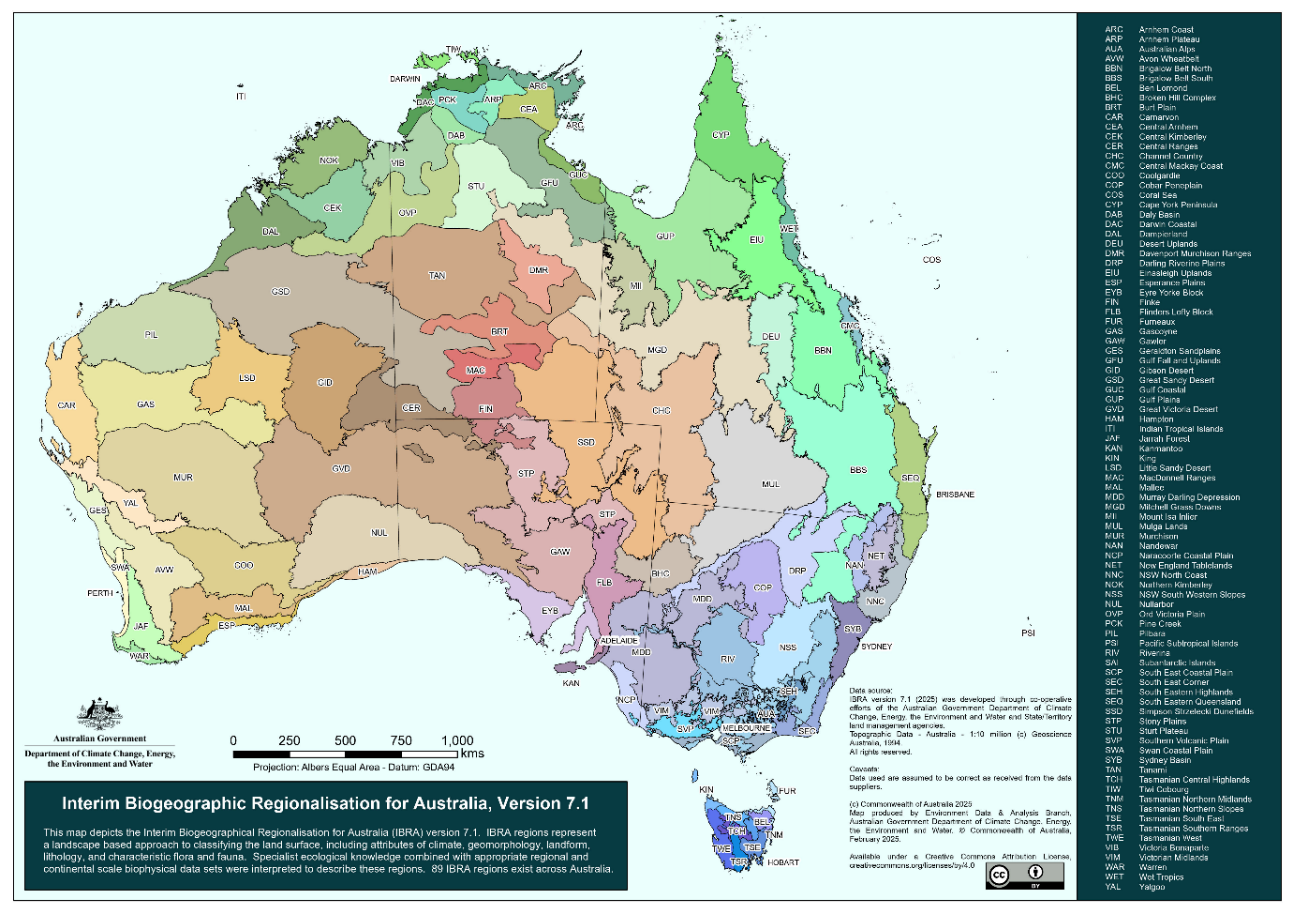


Supplementary Figure 1: (a) IBRA bioregions used for geographic orientation in identifying hotspots; (b) IBRA map version 7.1 from the Department of Climate Change‚ Energy‚ the Environment and Water (2025a)

Supplementary Table 1: Study sites where fieldwork was conducted.

| **Site** | **Latitude** | **Longitude** |
| --- | --- | --- |
| The Badger Ground* | -32.634768 | 149.977530 |
| Millewa Precinct, Murray Valley National Park (MVNP) | -35.800320 | 145.041102 |
| Boomanoomana Precinct (MVNP) | -35.956755 | 145.859345 |
| The Hell Hole Forest Road, Ngulin Nature Reserve | -31.345381 | 151.619831 |
| Butterleaf National Park | -29.533132 | 152.034603 |
| Boonoo Boonoo National Park | -28.804019 | 152.186102 |
| The Kangaroo Flat Road, Yarrowitch | -31.279966 | 151.991638 |
| The Boree Creek, Borenore Karst Conservation Reserve | -33.249995 | 148.935574 |
| Mullion Range State Conservation Area | -33.165320 | 149.185718 |
| The Jones Creek, Jones Creek Reserve | -32.156761 | 148.784498 |
| Warrumbungle National Park | -31.274167 | 148.996111 |
| The Yearinan Trail, Ukerbarley State Conservation Area | -31.220765 | 149.214032 |

* A property in Breakfast Creek, New South Wales 2849, Australia

Supplementary Table 2: Key output from the nine models

| Model | Regularisation multiplier | Iterations | Training AUC | Test AUC | AUC Standard Deviation | Regularized training gain | Test gain |
| --- | --- | --- | --- | --- | --- | --- | --- |
| Original | 1 | 5000 | 0.9846 | 0.9839 | 0.0012 | 11.3605 | 19.0198 |
|  | 1.5 | 5000 | 0.9835 | 0.983 | 0.0012 | 2.0708 | 5.5924 |
|  | 2 | 5000 | 0.9858 | 0.9856 | 0.0008 | 15.8981 | 23.8981 |
| Background | 1 | 1920 | 0.8904 | 0.8882 | 0.0032 | 1.1236 | 1.2585 |
|  | 1.5 | 2196 | 0.8934 | 0.8898 | 0.0032 | 1.1661 | 1.2997 |
|  | 2 | 1236 | 0.8815 | 0.8796 | 0.0033 | 1.0236 | 1.1796 |
| Density | 1 | 5000 | 0.9783 | 0.9779 | 0.0011 | 11.3605 | 19.0198 |
|  | 1.5 | 5000 | 0.9764 | 0.9754 | 0.0013 | 2.0708 | 5.5924 |
|  | 2 | 5000 | 0.9679 | 0.9669 | 0.0015 | 15.8981 | 23.8981 |

Supplementary Table 3: Official descriptions for the main soil type contributing to the modelling (National Resource Information Centre, 1991).

| **Soil unit** | **Description** |
| --- | --- |
| Mb2 | Dissected sandstone plateau of moderate to strong relief with sandstone pillars, ledges, and slabs level to undulating ridges, irregularly benched slopes, steep ridges, cliffs, canyons, narrow sandy valleys: chief soils are (i) on areas of gentle to moderate relief, acid yellow leached earths (Gn2.74) and (Gn2.34) and acid leached yellow earths (Gn2.24)-sometimes these soils contain ironstone gravel; and (ii) on, or adjacent to, areas of strong relief, siliceous sands (Uc1.2), leached sands (Uc2.12) and (Uc2.2), and shallow forms of the above (Gn2) soils. Associated are: (i) on flat to gently undulating remnants of the original plateau surface, leached sands (Uc2.3), siliceous sands (Uc1.2), sandy earths (Uc5.22), and (Gn2) soils as for (i) above (these areas are in part comparable with unit (Cb29); (ii) on flat ironstone gravelly remnants of the original plateau surface, (Gn2) soils as for unit Mb5(i); (iii) on gently undulating ridges where interbedded shales are exposed, shallow, often stony (Dy3.41), (Dr2.21), and related soils similar to unit Tb35; (iv) narrow valleys of (Uc2.3) soils flanked by moderate slopes of (Dy3.41) soils; (v) escarpments of steep hills with shallow (Dy) and (Dr) soils between sandstone pillars; and (vi) shallow (Um) soils, such as (Um6.21) on steep hills of basic rocks. |

| **Soil unit** | **Description** |
| --- | --- |
| Me1 | Hilly with some steep slopes and small graded valleys: moderately steep rounded hills of brown and red friable earths (Gn3.21, Gn3.22, Gn3.11, Gn3.12, and Gn3.14) in association with less rounded hill slopes of hard acidic yellow mottled soils (Dy3.41) and (Dy3.21), hard acidic red soils (Dr2.21), and yellow leached friable earths (Gn3.54), and also other hill slopes of loamy soils having an A2 horizon (Um4.2) with yellow-brown earths (Gn2.44); stream valleys of various soils including (Dy4.41) (Dy5.41), and (Um6.11). This unit is a broad one. In some areas the (Dy) and (Dr) soils with the (Um4.2) and the (Gn2.44) soils are dominant rather than the (Gn3) soils. |
| Mw1 | Flat to undulating range crests flanked by hilly to steep hilly terrain: range crests of acid leached red earths (Gn2.14) and possibly other undescribed soils. Associated are: hill slopes of sandy soils, such as (Uc3.32), with sandy acidic soils with clay subsoils, such as (Dr4.61) and (Dy5.61); and other, contrasting hill slopes of (D) soils with friable surface soils, such as (Dd3.21), (Db4.1), and (Dr4.1), and also yellow-brown earths (Gn2.44). Stream valleys are narrow with steep side slopes. |

| **Soil unit** | **Description** |
| --- | --- |
| Me2 | Steep hilly to mountainous with incised stream valleys: steep but more or less rounded hill slopes of brown friable earths (Gn3.21 and Gn3.22) and possibly some (Gn4) soils, in association with: at the higher altitudes, steep hill slopes of sandy soils (Uc4.2), loamy soils having an A2 horizon (Um4.2) with yellow-brown earths (Gn2.44), and possibly (Uc6.11) and (Um5.41) soils; and at the lower altitudes, moderate to steep slopes of hard acidic yellow mottled soils (Dy3 21 and Dy3.41), hard acidic red soils (Dr2.21), and yellow leached friable earths (Gn3.54); and narrow incised stream valleys of various soils including (Um6.11) and (Dy) soils. This unit is a broad one. In some areas the (Um4.2) and (Gn2.44) soils and in others the (Dy) and (Dr) soils rather than the (Gn3) soils could be dominant. |
| Pb4 | Mountainous at low to moderate elevation: moderate to steep hill slopes of hard acidic red soils (Dr2.21) with hard acidic yellow mottled soils (Dy3.21) and other (Dy) soils including (Dy2.2, Dy2.41, Dy2.71, Dy3.81) and (Dr) soils including (Dr2.61); in association with loamy soils with an A2 horizon (Um4.2 and Um4.1), shallow grey-brown sandy soils (Uc6.11), and rock outcrops; small areas of soils of unit Mh4 of Sheet 2 at the higher altitudes; small areas of (Dy3.41) soils in areas of restricted drainage at low altitudes; dissected by stream valleys, soils not described. As mapped on Sheet 3 (north of the Murray River), areas of unit Pf1 are included. |

| **Soil unit** | **Description** |
| --- | --- |
| LL1 | Hills and plains--multicyclic erosional landscape of hills and hillocky areas with intervening areas of dissected plains, the whole traversed and dissected by variously incised stream valleys. Some layering of soil materials: (1) relatively higher hills and ranges of loamy soils having an A2 horizon (Um4.2) and yellow-brown earths (Gn2.44) with (Um5.41 and Um5.51) many stones and rock outcrops; gullies of (Dr2) and (Dy3.32 and Dy3.42) soils; (2) relatively lower hills and hillocky areas of hard acidic red soils (Dr2.21) and (Uc6.11), (Um) soils and rock outcrops with (Dy3.4) soils on lower slopes and (Dy3.43) in depressions; (3) undulating plains with slopes and benches of red and yellow earths including (Gn2.12, Gn2.14, Gn2.15, and Gn2.24); (4) stream valleys of (Um6.11), some with clay D horizons and other (Uc) and (Um) soils; (5) also remains of various soil materials such as ironstone boulders in various situations. |
| Cb21 | Plains with some dunes; flat to undulating areas of leached sands (Uc2.33) with dunes of (Uc2.21) and (Uc2.33) and with swamps of (Um6.11) and acid peaty soils; traversed by streams with terraces and floodplains of (Uf) and (Um) soils; broken by a few low hills of (Uc4.11) soils. |

| **Soil unit** | **Description** |
| --- | --- |
| Mh5 | Rugged mountainous country at moderate to high elevation; soil variation is considerable with a general order as follows, moderate to steep mountain slopes of brown and red friable porous earths (Gn4.31, Gn4.34, and Gn4.14) with smaller areas of (Gn4.11) and/or red earths (Gn2.11 and Gn2.14) with smaller areas of yellow earths (Gn2.21 and Gn2.24); in association with (1) at the relatively higher altitudes, small areas of organic loamy soils (Um7.11) or shallow grey-brown sandy soils (Uc6.11) or loamy soils with an A2 horizon (Um4.2); and (2) at the relatively lower altitudes, moderate areas of friable acidic red soils (Dr4.21) and friable acidic yellow soils (Dy4.21, Dy4.61, Dy5.21) or friable brown soils (Db3.11); and (3), in the drier situations at the relatively lower altitudes, small areas of hard acidic red soils (Dr2.21) and hard acidic yellow mottled soils (Dy3.21); and (4) narrow stream valleys with small floodplains of variable soils, largely undescribed, but including (Um5.5), (Ug6.1), and (Dy5.42). |
